# Undergraduate Global Health Degrees: The Time is Right

**DOI:** 10.4269/ajtmh.16-0835

**Published:** 2017-01-11

**Authors:** Timothy F. Brewer

**Affiliations:** 1 UCLA Office of the Vice Provost, Interdisciplinary and Cross Campus Affairs, Los Angeles, California.

In this issue of the journal, Drain and others^1^ report on one of the newest areas of global health growth, baccalaureate degrees for college and university undergraduates. Although global health certificate programs have been rapidly expanding, some experts and university leaders have been ambivalent about whether the field has developed enough to warrant offering majors.

Global health issues increasingly command the consideration of everyone up to presidents and prime ministers^2^; they deserve universities' attention. Though just under 800 fatal severe acute respiratory syndrome cases were reported during the 2002–2003 outbreak, or approximately the same number of deaths as occur in Los Angeles County every 5 days, this epidemic resulted in $30 to $50 billion in economic losses globally.^3^ Today, pregnant women throughout the Western Hemisphere live in fear of Zika virus, which until a few years ago was responsible for an obscure disease assumed to cause inconsequential infections.

At least three potentially paradigm shifting opportunities exist for undergraduate global health education. First is the chance to substantially expand the pool of practitioners. Approximately 31,500 students graduated from U.S. medical schools or with a public health graduate degree in 2015^4^,^5^; just over 2.8 million students graduated from a U.S. college or university with a bachelor's or higher degree.^6^ Attracting even a fraction of the percentage of medical and public school graduates who enter global health careers from among new university graduates to work in global health would considerably expand the available workforce.

Engaging experts from disciplines beyond the health sciences to pursue global health careers could be a second truly transformative possibility. Duke and New York University require global health majors to have a second degree in another discipline; 15% at Duke chose the Humanities. Arizona State University's global health program is based in the School of Human Evolution and Social Change, whereas the University of California, San Diego's program is run through the Department of Anthropology.^1^ What relevance might a liberal arts education have for achieving a world free from tropical infectious diseases? After all, the health sciences have made remarkable contributions to reducing the burden of infectious diseases and expanding human life expectancy over the last 100 plus years.^7^,^8^

A few years ago, Farmer and Ivers asked in the pages of this journal how cholera, “a scourge against which we have a full arsenal of preventatives and therapeutics continue[s] to fell hundreds of thousands of people every year?”^9^ More recently, the failure to stop Ebola virus transmission in Guinea, Sierra Leone, and Liberia during the latter half 2014 and into 2015 resulted in a humanitarian catastrophe. Neither the magnitude nor the duration of the cholera outbreak in Haiti or the Ebola outbreak in west Africa, however, were fundamentally due to a lack of knowledge regarding the biology or epidemiology of the pathogens or the prevention and control steps necessary to contain them. Rather, it was the global community's inability or unwillingness to apply its collective health expertise within the specific cultural and socioeconomic contexts in which these outbreaks were occurring that resulted in thousands of lives unnecessarily lost.

Why did the global health community fail to curtail these outbreaks, leaving affected communities still dealing with the consequences when other outbreaks were more rapidly contained, including a simultaneous Ebola outbreak in the Democratic Republic of Congo? Plasmodia from the same species reasonably can be expected to behave in a similar fashion under comparable conditions, but people are not protozoa. Individual and collective hope, fear, compassion, jealousy, or cultural heritage do not influence microbial behavior; the same cannot be said for us. Health expertise was a necessary, but not sufficient, tool for controlling these crises in a timely manner.

Previous presidents of the American Society of Tropical Medicine and Hygiene have called on the society members to address gender inequality and poverty, correctly recognizing that these factors are major contributors to disease morbidity and mortality globally.^7^,^10^ But there is no biomedical solution for poverty or gender inequality. If our goal is a world free from tropical infectious diseases, then partners from other disciplines—not just engineers or lawyers, but philosophers, artists, anthropologists, and historians—are needed to help address these and other key health determinants. It is difficult to image a National Institutes of Health study section funding the preservation of ancient texts of Chinese herbal medicine; yet, few discoveries in recent decades have had as profound an impact on health globally as that made by Youyou Tu and others from studying those texts.^11^

Finally, a public more knowledgeable about global health issues could be a third notable benefit of these programs. Though only a minority of majors may end up in global health careers, challenges such as the human immunodeficiency virus pandemic or the Ebola virus 2014–2015 outbreak have widespread societal effects. Agencies not traditionally associated with health, including the U.S. State Department, the World Bank, and the United Nations Security Council have become critical actors in responding to global health crises. An informed public and political leadership, exposed to global health issues as undergraduates, could become our most valuable advocates for programs and resources to address these challenges.

Although the health sciences continue to probe deeper into understanding the myriad forms of life, the liberal arts, arts, social sciences, and other disciplines help us comprehend what it means to be alive. Undergraduate programs open opportunities for talented students from diverse disciplines to ask and to seek answers to questions fundamental for achieving equitable health worldwide, or to become lifelong global health advocates. We should encourage and support universities' efforts to establish global health degree programs. After all, it will not just be our capacity to understand the pathogens that share our world that ultimately determines whether we achieve health globally, but our ability to understand ourselves.
